# Is SARS-CoV-2 responsible for relapses of Parkinson’s disease?

**DOI:** 10.1186/s41983-021-00344-x

**Published:** 2021-07-02

**Authors:** Fulvio A. Scorza, Josef Finsterer, Ana C. Fiorini

**Affiliations:** 1 Disciplina de Neurociencia, Universidade Federal de Sao Paulo/Escola Paulista de Medicina (UNIFESP/EPM), Rua Pedro de Toledo, 697-Vila Clementino, Sao Paulo, 04039-00 Brazil; 2 Klinik Landstrasse, Messerli Institute, Postfach 20, 1180 Vienna, Austria; 3 Programa de Estudos Pos-Graduado em Fonoaudiologia, Pontifícia Universidade Catolica de Sao Paulo (PUC-SP), Sao Paulo, Brazil; 4 Departamento de Fonoaudiologia, Escola Paulista de Medicina/Universidade Federal de Sao Paulo (EPM/UNIFESP), R. Botucatu, 740-Vila Clementino, Sao Paulo, 04023-062 Brazil

**Keywords:** SARS-CoV-2, COVID-19, Parkinson’s disease, Complications, Movement disorder

With interest, we read the article by Kamel et al. about a 56-year-old male with Parkinson’s disease (PD) in whom worsening of PD was presented as the exclusive clinical manifestation of a SARS-CoV-2 infection [1]. The study is appealing but has several limitations which raise comments and concerns.

The major limitation of the study is that various causes for the deterioration of PD were not excluded. No cerebral imaging had been carried out when PD deteriorated. Mechanical ventilation is no contra-indication for cerebral imaging. Missing are also a positron emission tomography (PET) study respectively a dopamin transporter (DAT) scan. No investigations of the cerebro-spinal fluid (CSF) had been carried out to exclude encephalitis or meningitis.

The patient was confused on admission; thus, it is conceivable that there was mismanagement of his medication and that he had developed side effects of overdosages, such as the dopamin (DA) agonist, as he had previously experienced. We should be told for how long the patient was confused already at home and if he was responsible for the management of his daily medication alone or his caregivers.

Missing is the information about the cause of respiratory distress requiring mechanical ventilation. We should be told if respiratory distress resulted from a central nervous system (CNS) cause, from muscular compromise, or from pulmonary infection, in particular COVID-19 pneumonia. Since muscular respiratory distress could be also due to Guillain-Barre syndrome (GBS), increasingly recognised as a complication of SARS-CoV-2 infections [2], it is crucial that the index patient had undergone appropriate CSF investigations and nerve conduction studies.

A further limitation is that it is not reported how worsening of PD was managed. We should be told if the levodopa carbidopa dosage was increased or if the dosage of the DA-agonist was increased. Since the authors speculate that the infection could be responsible for increased levodopa requirement, it is not comprehensible why he was maintained on his regular dosage [1].

The authors speculate that CNS affection by the virus could be responsible for deterioration of PD but do not provide evidence for this speculation. No evidence was provided that the virus had entered the CNS at all or that there was an immune response mainly affecting the CNS.

We do not agree that ‘genetic PD makes the patient vulnerable to immunologically mediated neuronal damage’ [1]. This statement is not substantiated by any evidence. Why should immune mechanisms affect patients with genetic and non-genetic PD differentially?

Overall, this interesting case has several limitations which challenge the results and their interpretation. Since the cause of PD deterioration remained undetermined, establishing a causal relation between the SARS-CoV-2 infection and the relapse of PD is not justified. Since deterioration of PD can be multicausal, work-up and exclusion of all these differential causes are warranted.

## Data Availability

All data reported are available from the corresponding author.

## Abbreviations

CNS: Central nervous system
CSF: Cerebro-spinal fluid
DA: Dopamin
DAT: Dopamin transporter
GBS: Guillain-Barre syndrome
PD: Parkinson’s disease
PET: Positron emission tomography
